# Environmental surveillance of pathogens in Africa

**DOI:** 10.1128/aem.01932-25

**Published:** 2026-04-29

**Authors:** Eric Darko, Debora Akortia, Gifty Nkrumah, Francis Opoku Agyapong, Sampson Twumasi-Ankrah, Michael Owusu-Ansah, Rosanna Glazik, Alexander Shaw, Nicholas Grassly, Ellis Owusu-Dabo, Yaw Adu-Sarkodie, Michael Owusu

**Affiliations:** 1 Department of Clinical Microbiology, Kwame Nkrumah University of Science and Technology 98763, Kumasi, Ghana; 2 Department of Statistics and Actuarial Science, Kwame Nkrumah University of Science and Technology 98763, Kumasi, Ghana; 3 School of Public Health, Kwame Nkrumah University of Science and Technology 98763, Kumasi, Ghana; 4 University of Warwick 2707, Coventry, United Kingdom; 5 Department of Infectious Disease Epidemiology, School of Public Health, MRC Centre for Global Infectious Disease Analysis, Imperial College London 156430, London, United Kingdom; 6 Department of Medical Diagnostics, Kwame Nkrumah University of Science and Technology 98763, Kumasi, Ghana; The Pennsylvania State University, University Park, Pennsylvania, USA

**Keywords:** environmental surveillance, pathogens, rapid review

## Abstract

The control of infectious diseases depends on effective diagnostics and interventions. In Africa, resource limitations hinder clinical surveillance. Environmental surveillance (ES), particularly wastewater surveillance, offers a cost-effective alternative. While globally expanding, its application in Africa remains limited. This review aimed to describe published studies in Africa that have utilized ES for the detection of infectious pathogens of public health importance in Africa. The study employed a rapid review approach to synthesize evidence on ES of infectious pathogens in Africa, following guidance from the Cochrane Rapid Reviews Methods. Articles from major databases, including Scopus, PubMed, Science Direct, and Cochrane, were screened using Catchii.org. Duplicates were removed, and data were extracted into Excel, and study quality was appraised using the AXIS tool and a modified Newcastle-Ottawa Scale. The search strategy identified 2,189 articles, of which 90 were found to be eligible. We identified 47 microbial species that have been reported across studies. These consisted of 46.8% bacteria (*n* = 22), 36.2% viruses (*n* = 17), 4.3% fungi (*n* = 2), and 12.7% parasites (*n* = 6). Among viruses identified, SARS-CoV-2 was the most common, followed by rotaviruses and polioviruses. *Vibrio cholerae* was mostly reported among bacterial pathogens. The most common sampling method was grab sampling (*n* = 85, 94.4%), while two-phase separation (*n* = 22, 37.3%) and filtration (*n* = 11, 39.3%) were the most frequently used concentration methods for viral and bacterial detection, respectively. ES of infectious pathogens in Africa remains limited. There is a need to expand this to enhance pathogen monitoring, transmission insights, and preparedness for emerging variants.

## BACKGROUND AND RATIONALE

Disease pandemics and epidemics have led to an increase in morbidity and mortality in the world, especially in Africa (1). The economic crises and healthcare challenges have greatly contributed to the high disease burden in Africa (2). Containment of infectious diseases depends largely on the of diagnostic methods and sustainable, effective interventions. The cost of disease diagnosis is a major concern for most African countries, especially sub-Saharan countries (3). The region continues to experience outbreaks and epidemics of infectious diseases, often exacerbated by poor health systems and inadequate diagnostic infrastructure, late detection, and ineffective outbreak response (4). The inability to diagnose an infection may lead to the spread of the pathogen, making it difficult to contain the infection at the community, national, and world levels (5).

The achievement of Sustainable Development Goal 6 (SDG 6), a United Nations target aimed at ensuring the availability and sustainable management of water and sanitation for all, remains challenging in Africa due to widespread environmental pollution and contamination (6, 7). The environment plays a vital role in the life cycle of infectious agents. Therefore, techniques capable of detecting infectious agents in environmental matrices can support the assessment of disease burden associated with specific pathogens (8). Studies have recommended an environmental surveillance (ES) approach for low- and middle-income countries to complement existing clinical surveillance in determining disease burden (8–10). The ES approach is relatively cost-effective, assists in the early detection of pathogens, and provides an understanding of the transmission dynamics of pathogens in the environment (8).

ES aligns closely with Africa’s Integrated Disease Surveillance and Response (IDSR) system by complementing case-based surveillance through the early detection of pathogens at the population level, particularly in settings where underreporting and limited diagnostic access constrain routine surveillance (11). By generating timely environmental signals of pathogen circulation independent of healthcare utilization, ES supports IDSR objectives of early warning and rapid response. In addition, ES is consistent with the One Health framework, as it captures pathogen dynamics at the human environment interface (12). Integrating ES into IDSR and One Health-informed surveillance structures can strengthen cross-sectoral collaboration, enhance outbreak preparedness, and improve the overall effectiveness of infectious disease surveillance systems in Africa (12).

The detection and monitoring of infectious agents in environmental samples provide knowledge on the epidemiology of pathogens of waterborne, respiratory, enteric, and other transmission routes (8, 13). Surveillance of pathogens in environmental samples helps to predict potential disease outbreaks in communities. Methods used in ES of pathogens vary greatly spanning sample collection, laboratory processing, and detection of target pathogens (14). The choice of methods for ES studies depends largely on the cost involved, implementation challenges, and effective detection of target pathogens. Several studies across Africa have independently investigated the presence of various infectious agents in environmental samples, including poliovirus (15–19), norovirus (20), SARS-CoV-2 (21, 22), *Salmonella* Typhi (14, 23), cryptococci (24, 25), and *Vibrio cholerae* (26, 27). ES of pathogens in Africa has also been reviewed in papers that focused primarily on a single infectious agent at a time, such as polioviruses (28) and SARS-CoV-2 (22) or *Salmonella* Typhi (14). The potential for multi-pathogen surveillance in the environment has not been well described in previous reviews. A review that synthesizes ES studies detecting different and multiple pathogens across diverse environmental samples and matrices provides a broader platform for understanding the scope and diversity of environmental surveillance activities in Africa, in contrast to previous reviews that focused predominantly on single pathogens.

The review aimed to assess the frequency and distribution of published ES studies in Africa, infectious pathogens detected, and laboratory methods for concentrating samples and detection techniques. Specifically, the review identifies and describes the available ES studies on microbial pathogens, including viruses, bacteria, fungi, and parasites, the countries where such studies have been conducted, and the predominant sampling, concentration, and detection methods used.

## REVIEW FRAMEWORK AND LITERATURE SEARCH

### Review design and scope

The study employed a rapid review methodology to synthesize existing evidence on the application of environmental surveillance for detecting infectious pathogens in Africa. The review was conducted in accordance with rapid review guidance from the Cochrane Rapid Reviews Methods Group (29), with methodological adaptations made to expedite the process while maintaining scientific rigor.

The review focused on peer-reviewed papers that reported the use of ES methods to detect and monitor bacterial, viral, parasitic, and fungal pathogens in environmental matrices such as wastewater and surface water within African countries. The primary objective was to assess the extent and scope of environmental surveillance efforts targeting infectious agents across the continent. To address this aim, the following specific objectives were outlined:

- To identify and map African countries where environmental surveillance has been conducted to assess the burden of infectious diseases.
- To determine the types of infectious pathogens (bacterial, viral, parasitic, and fungal) detected through environmental surveillance in Africa.
- To characterize the sampling strategies, concentration methods, and detection techniques employed in environmental surveillance studies for pathogen detection in wastewater and other environmental matrices.

### Inclusion criteria

Peer-reviewed articles published in the English language were eligible to be part of this study, and no limitation was set on the publication year of the papers. Articles on environmental surveillance studies (using wastewater and surface water) conducted in Africa on infectious agents such as viruses, bacteria, fungi, and parasites were included in the review.

### Exclusion criteria

The review did not include environmental surveillance studies conducted outside Africa. Articles that used non-environmental samples, such as non-wastewater and non-surface water (treated water, groundwater from boreholes, rainwater collected for domestic use, industrial process water, and soils), were also excluded. ES studies focusing primarily on antimicrobial resistance (AMR) genes in environmental samples, without linkage to specific pathogenic organisms, as well as studies related to animal or pharmaceutical surveillance, were excluded. In contrast, ES studies reporting the detection of identifiable pathogens, including those that additionally characterized associated AMR genes, were included.

### Sources of information and literature search strategy

The review used published articles from Scopus, PubMed, ScienceDirect, and Cochrane. Keywords and phrases such as “environmental surveillance,” “wastewater epidemiology,” “wastewater surveillance,” “Africa,” “African countries,” “microbes,” “pathogens,” “infectious agents,” “sampling methods,” “laboratory methods,” “concentration techniques,” and “detection methods” were searched on the database platforms to generate articles related to the focus and objectives of the study. A modified Population, Intervention, Control, and Outcome (PICO) framework was used to streamline the boundaries of the research questions during the search strategy. The PICO framework used for searching articles from the various databases is described in Table 1. Generated articles from the databases were imported into the Catchii software (https://app.catchii.org/) for the removal of duplicates and the subsequent screening of articles. The period for the search strategy, screening of articles, and data extraction for the review was conducted from October 2024 to November 2024.

**TABLE 1:** Search strategy of articles from online electronic databases

Search no.	PICO parameters	Descriptions	Results
Scopus			
# 1	Populations	“African countries” OR Africa	2,660,855
# 2	Intervention	(“wastewater surveillance”) OR (“environmental surveillance”)	10,576
# 3	Outcome	“infectious pathogens” OR “infectious agents” OR bacterium OR viruses OR parasite OR fungi	7,276,658
	#1 and #2 and #3	(“African countries” OR Africa) AND (“wastewater surveillance” OR “environmental surveillance”) AND (“infectious pathogens” OR “infectious agents” OR bacteria OR viruses OR parasite OR fungi)	1,360
PubMed			
# 1	Populations	Algeria or Angola or Benin or Botswana or Burkina Faso or Burundi or Cape Verde or Cameroon or Central African Republic or Chad or Comoros or Congo or Democratic Republic of the Congo or Côte d'Ivoire or Ivory Coast or Djibouti or Egypt or Equatorial Guinea or Eritrea or Eswatini or Ethiopia or Gabon or Gambia or Ghana or Guinea or Guinea-Bissau or Kenya or Lesotho or Liberia or Libya or Madagascar or Malawi or Mali or Mauritania or Mauritius or Morocco or Mozambique or Namibia or Niger or Nigeria or Rwanda or São Tomé and Príncipe or Senegal or Seychelles or Sierra Leone or Somalia or South Africa or South Sudan or Sudan or Tanzania or Togo or Tunisia or Uganda or Zambia or Zimbabwe	73,952
# 2	Intervention	(wastewater surveillance) OR (environmental surveillance)	18,636
# 3	Outcome	(viruses or bacteria or fungi or parasites) OR (infectious pathogen) OR (infectious agents) OR (pathogens)	464,292
	(#1) and (#2) and (#3)	(Algeria or Angola or Benin or Botswana or Burkina Faso or Burundi or Cape Verde or Cameroon or Central African Republic or Chad or Comoros or Congo or Democratic Republic of the Congo or Côte d'Ivoire or Ivory Coast or Djibouti or Egypt or Equatorial Guinea or Eritrea or Eswatini or Ethiopia or Gabon or Gambia or Ghana or Guinea or Guinea-Bissau or Kenya or Lesotho or Liberia or Libya or Madagascar or Malawi or Mali or Mauritania or Mauritius or Morocco or Mozambique or Namibia or Niger or Nigeria or Rwanda or São Tomé and Príncipe or Senegal or Seychelles or Sierra Leone or Somalia or South Africa or South Sudan or Sudan or Tanzania or Togo or Tunisia or Uganda or Zambia or Zimbabwe) AND ((wastewater surveillance) OR (environmental surveillance)) AND ((viruses or bacteria or fungi or parasites) OR (infectious pathogen) OR (infectious agents) OR (pathogens))	461
ScienceDirect			
# 1	Populations	“African countries”	338,181
# 2	Intervention	“environmental surveillance” OR “wastewater surveillance” OR “wastewater epidemiology”	2,767
# 3	Outcome	“infectious pathogens” OR “infectious agents” OR bacterium OR viruses OR parasite OR fungi	>1,000,000
	#1 and #2 and #3	(African countries) AND (“environmental surveillance” OR “wastewater surveillance”) AND (“infectious pathogens” OR “infectious agents” OR bacterium OR viruses OR parasite OR fungi)	353
Cochrane			
# 1a	Populations	(African countries) OR Algeria or Angola or Benin or Botswana or Burkina Faso or Burundi or Cape Verde or Cameroon or Central African Republic or Chad or Comoros or Congo or Democratic Republic of the Congo or Côte d'Ivoire or Ivory Coast or Djibouti or Egypt or Equatorial Guinea or Eritrea or Eswatini or Ethiopia or Gabon or Gambia or Ghana or Guinea or Guinea-Bissau or Kenya or Lesotho or Liberia or Libya or Madagascar or Malawi or Mali or Mauritania or Mauritius or Morocco or Mozambique or Namibia or Niger or Nigeria or Rwanda or São Tomé and Príncipe or Senegal or Seychelles or Sierra Leone or Somalia or South Africa or South Sudan or Sudan or Tanzania or Togo or Tunisia or Uganda or Zambia or Zimbabwe	27,962
# 1b		MeSH descriptor: (Africa) explode all trees	12,737
		#1a OR #1b	28,196
# 2a	Intervention	(environmental NEXT surveillance): OR (“environmental surveillance” OR (wastewater NEXT (surveillance* or epidemiology*): ti,ab,kw) OR (sewage NEXT surveillance*)	8
# 2b		MeSH descriptor: (environmental monitoring) explode all trees	499
		#2a OR #2b	505
# 3a	Outcome	(*infectious NEXT (pathogen* or agent*)) OR (pathogen OR bacteria OR viruses OR parasites OR fungi)	71,045
#3b		MeSH descriptor: (virulence) explode all trees	72
		#3a OR #3b	71,051
	#1 and #2 and #3	(African countries) AND (“environmental surveillance” OR “wastewater surveillance”) AND (“infectious pathogens” OR “infectious agents” OR bacterium OR viruses OR parasite OR fungi)	353
		(#1a OR #1b) AND (#2a OR #2b) AND (# 3a OR #3b)	15

### Study or article selection

ES studies conducted in Africa on infectious agents such as viruses, bacteria, fungi, and parasites were selected to be part of the review. The review also considered papers that highlighted countries in Africa that have used environmental surveillance approaches to determine the pathogen-disease burden in their communities.

The study screened for articles that used sampling techniques and concentration methods employed in ES studies. Screening of papers for the review also focused on data-evidence articles for estimating disease burden and implementing intervention strategies such as vaccination. Papers that provided findings on the transmission dynamics of the ES pathogen in communities were selected during the screening stage. Improvement of water and sanitation hygiene properties based on evidence from ES studies was selected, as such papers helped evaluate the performance of countries in achieving SDG 6. The details of the inclusion and exclusion criteria are highlighted below.

### Screening process and confirmation of articles

After detection, duplicate articles were removed, and initial screening was carried out by two reviewers (E.D. and F.O.A.) independently, considering the title and abstract of the articles with reference to the keywords and phrases for the review paper. Decisions on the screened articles were made, confirmed, and proceeded to determine their eligibility.

**Fig 1:**
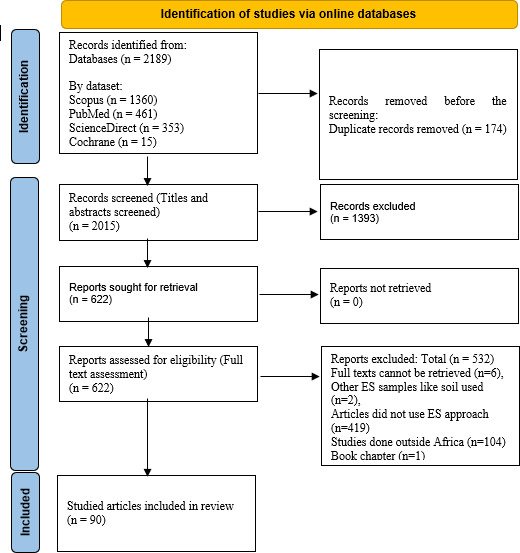
PRISMA flow diagram.

The full texts of the resultant articles were analyzed independently by two reviewers (E.D. and F.O.A.) to determine their eligibility to be part of the review. Articles that could not be fully accessed were excluded. Additionally, articles that did not respond to the study’s objectives or could not meet the inclusion criteria after assessing the full text were excluded. Eligible articles were confirmed and included in the review. The history of all decisions and changes made during the screening process and the determination of eligible articles can be found in an attached document (Excel format). Article identification, screening, and eligibility stages are displayed in the Preferred Reporting Items for Systematic Reviews and Meta-Analyses (PRISMA) flow diagram, which can be seen in Fig. 1.

### Data extraction

The extraction of data from the included articles was done after the screening process, and confirmation decisions were made. Data from the eligible articles relevant to the review’s objectives were extracted and documented in the Excel document attached. The extracted data were entered into the spreadsheet using the following subheadings: article title, article type, journal name, country of study, period of study, date of publication, type of infectious agent studied, name of infectious agent, study design type, sample type, sample size, sampling method, laboratory processing method, and method of detection. The subheadings were previously discussed and revised among all the authors. Authors E.D. and F.O.A. abstracted the data from the included articles, and this was supervised by co-author M.O. Errors and discrepancies were resolved by E.D. and F.O.A. Several co-authors reviewed the final data for completeness and accuracy. A copy of the extracted data for the review can be found in the attached document (Excel format).

### Data synthesis

Relevant data were extracted from eligible articles, and descriptive analysis was performed. Infectious agents detected in environmental samples from eligible articles were grouped into bacteria, viruses, fungi, and parasites. The transmission means of these pathogens, such as respiratory and fecal-oral routes, are reflected in the categorization and discussion of the infectious pathogens detected in ES samples.

Sample collection, concentration, and detection techniques of pathogens from eligible articles were grouped and reported. Data on sample types (wastewater, sewage, and surface water) and sampling techniques (Moore swab and grab water) used in ES studies were collated and discussed. Laboratory concentration methods and detection techniques from the selected articles were highlighted and discussed. Countries in Africa where ES studies have been published were identified and collected for a narrative synthesis. Prevalences of pathogens of interest recorded from included studies were identified and used to provide pooled prevalence values for the types of infectious agents and frequently detected specific pathogens in this review.

### Quality assessment of data

The quality of the articles included in the review was assessed to determine the risk of bias. Modified Newcastle Ottawa Scale (NOS) was used to assess articles with a longitudinal study design (studies with more than one sample collected per site for a period interval of at least 48 hours), focusing on three major aspects: selection of the study group, comparability of the groups, and outcome. The modified NOS consisted of 10 questions, and values or stars recorded were interpreted as from 0 to 9, with studies rating 0–3 (poor quality), 4–6 (fair quality), and 7–9 (good/high quality) (30, 31).

Articles with cross-sectional designs (studies with one-point time sampling) were assessed using the Appraisal tool for Cross-Sectional Studies (AXIS). The AXIS comprised 18 questions with a focus on introduction, methods, results, and discussion. The assessment of the data was done by authors E.D., D.A., and G.N. (32). The detailed quality assessment of articles with both longitudinal and cross-sectional study designs is provided in the Miscellaneous File.

## CURRENT LANDSCAPE OF ENVIRONMENTAL SURVEILLANCE RESEARCH IN AFRICA

### Overview of screening processes and characteristics of the included articles

The search strategy (Table 1) conducted from October 2024 to November 2024 resulted in 2,189 articles from online databases comprising Scopus (*n* = 1,360), PubMed (*n* = 461), ScienceDirect (*n* = 353), and Cochrane (*n* = 15). All the generated articles were uploaded into the catchii.org review platform, and 174 duplicates were detected and removed. The titles and abstracts of the remaining 2,015 articles were screened (initial screening), resulting in the exclusion of 1,393 articles, leaving 622 articles for full-text screening. A total of 532 articles were found ineligible to be included in the review during the full-text screening. Reasons for the exclusion of the articles and other details of the screening processes are presented in the PRISMA flowchart in Fig. 1. Data were extracted from 90 peer-reviewed eligible articles, comprising 87 original research articles (96.7%) and 3 brief reports (3.3%).

Articles included in this review were published from 1991 to 2024, with most of the articles published during and after the COVID-19 pandemic (*n* = 59, 65.6%). Periods of studies for the eligible articles ranged from 4 days to 9 years, with a higher number of articles conducted in a longitudinal study design (*n* = 83, 92.2%), of which 81.9% (*n* = 68) are of good quality assessment compared to a cross-sectional design (*n* = 7, 7.8%) of which all were of good quality. Sample sizes in the reviewed studies varied widely, ranging from 4 to 1,127 samples. Wastewater was the predominant sample type used (*n* = 43, 47.8%), followed by surface water samples (*n* = 26, 28.9%) and sewage samples (*n* = 16, 17.8%). The combined use of sewage and wastewater was the least reported sampling type, appearing in five studies (5.5%).

### Study findings

#### African countries with ES studies and pathogens detected

A total of 47 distinct microbial species were identified in the 90 eligible articles. Among the microbial species detected, bacteria accounted for the highest proportion at 46.8% (*n* = 22), followed by viruses at 36.2% (*n* = 17), parasites at 12.7% (*n* = 6), and fungi at 4.3% (*n* = 2). Viruses were the most commonly detected pathogen among the included articles reported in 59 studies (65.6%), followed by bacteria in 28 studies (31.1%), parasites in 5 studies (5.6%), and fungi in 2 studies (2.2%). These proportions may exceed 100% because several studies reported the detection of more than one microbial group and were therefore counted in multiple categories.

Among the studies that reported on viruses (*n* = 59), SARS-CoV-2 was the most frequently detected pathogen, identified in 14 (23.7 %) articles, followed closely by rotavirus in 13 (22.0%) articles and poliovirus in 12 (20.3%) articles. Among the eligible articles that focused on bacterial pathogens, *Vibrio cholerae* was the most commonly detected species, reported in 11 (39.3%) articles, followed by *Escherichia coli* (*n* = 7, 25.0%) and *Salmonella* Typhi (*n* = 5, 17.9%). For parasitic pathogens, *Cryptosporidium* was detected in two studies (40.0%). Fungal pathogens were less commonly reported, with *Candida albicans* and *Cryptococcus* each detected in one study (50.0%). A detailed summary of pathogen detection across eligible studies is provided in Table 2.

**TABLE 2:** African countries with ES studies and some infectious pathogens detected^*a*^

African countries	Number of eligible articles (*N* = 90)	Type of pathogen (number of eligible articles)	Pathogens of key public health interest that were identified
Southern part of Africa	(*n* = 34)		
South Africa	24 articles	Bacteria (*n* = 9), viruses (*n* = 14), and parasites (*n* = 1)	SARS-CoV-2 (33–35), norovirus (35, 36), poliovirus (36, 37), rotavirus (38), *Vibrio cholerae* (39–41), *Mycobacterium tuberculosis* (42, 43), *Escherichia coli* (44)
Zimbabwe	2 articles	Viruses (*n* = 1) and bacteria (*n* = 1)	Enterovirus (45) and *Escherichia* c*oli* (46)
Mozambique	1 article	Viruses (*n* = 1)	Enterovirus (47)
Madagascar	2 articles	Viruses (*n* = 2)	SARS-CoV-2 (48) and poliovirus (49)
Zambia	2 articles	Viruses (*n* = 2)	SARS-CoV-2 (50), adenovirus (51), and rotavirus (51)
Malawi	3 articles	Bacteria (*n* = 3)	*Salmonella* Typhi (23, 44) and *Vibrio cholerae* (52)
North Africa	19 articles		
Egypt	13 articles	Viruses (*n* = 12), bacteria (*n* = 1), parasite (*n* = 2), and fungi (*n* = 1)	Poliovirus (53), Rotavirus (54, 55), *Salmonella* Typhi (56), *Shigella* (56), and *Candida albicans* (55, 56)
Tunisia	6 articles	Viruses (*n* = 6)	Enterovirus (*n* = 2) (57, 58), rotavirus (*n* = 1) (59), astroviruses (*n* = 1) (60), and SARS-CoV-2 (*n* = 3) (61, 62)
East Africa	15 articles		
Ethiopia	1 article	Viruses (*n* = 1)	SARS-CoV-2 (63)
Tanzania	4 articles	Bacteria (*n* = 3) and parasites (*n* = 1)	*Vibrio cholerae* (64), *Schistosoma haematobium* (65), and *Escherichia coli* (66)
Kenya	6 articles	Viruses (*n* = 5) and parasites (*n* = 1)	Poliovirus (67, 68), *Schistosoma mansoni* (69), rotavirus (70), and adenovirus (71)
Uganda	4 articles	Bacteria (*n* = 3) and viruses (*n* = 1)	*V. cholerae* (26, 72), *E. coli* (73), adenovirus (74), and enterovirus (74)
West Africa	23 articles		
Senegal	5 articles	Viruses (*n* = 5)	Poliovirus (16), non-poliovirus enteroviruses (NPEVs) (75), Aichi virus (76), SARS-CoV-2 (77), and human norovirus (77)
Ghana	8 articles	Viruses (*n* = 5) and bacteria (*n* = 3)	NPEV (19), poliovirus (78), SARS-CoV-2 (21), infectious spleen and kidney necrosis virus (79), *Escherichia coli* (80), *Klebsiella pneumoniae* (81), and *Vibrio cholerae* (82)
Cameroon	4 articles	Bacteria (*n* =1) and viruses (*n* = 3)	*V. cholerae* (27), poliovirus (40) HEV (54), and SARS-CoV-2 (48)
Nigeria	5 articles	Viruses (*n* = 2) and bacteria (*n* = 3)	Poliovirus (17), NPEV (83), *V. cholerae* (84), and *Acinetobacter baumannii* (85)
Burkina Faso	1 article	Bacteria (*n* = 1)	*Salmonella* (49) and *S. aureus* (86)

^*a*^ One article was conducted in two different countries (Cameron and Madagascar) (48).

Data from this review showed that environmental surveillance studies have been conducted across multiple regions of Africa, including North, East, West, and Southern Africa. A total of 17 African countries (31.5%) were represented in the eligible articles. Southern Africa accounted for the highest number of countries (*n* = 6) and contributed the largest proportion of studies (*n* = 34, 37.8%). West Africa followed with 5 countries, accounting for 23 (25.6%) studies. North Africa (2 countries) and East Africa (4 countries) contributed 19 (21.1%) and 15 (16.7%) studies, respectively. One eligible study was conducted across two countries (Cameroon and Madagascar) located in different regions of Africa and was therefore counted in both regions, accounting for the total of 91 studies included in the regional analysis. Data from the eligible articles showed that South Africa had the highest number of studies (*n* = 24, 26.7%), followed by Egypt (*n* = 13, 14.4%), Ghana (*n* = 8, 8.9%), and Kenya (*n* = 6, 6.7%).

Among the infectious pathogens, SARS-CoV-2 and rotavirus were detected in most of the articles conducted in South Africa (*n* = 5, 20.8%) and Egypt (*n* = 6, 46.2%), respectively. In Kenya, poliovirus (*n* = 2, 33.3%) and rotavirus (*n* = 2, 33.3%) were the pathogens detected in most of the included studies. Both poliovirus and non-poliovirus enteroviruses (NPEVs) were the viral pathogens with the highest number of articles reported in Ghana (*n* = 2, 25.0%).

**Fig 2:**
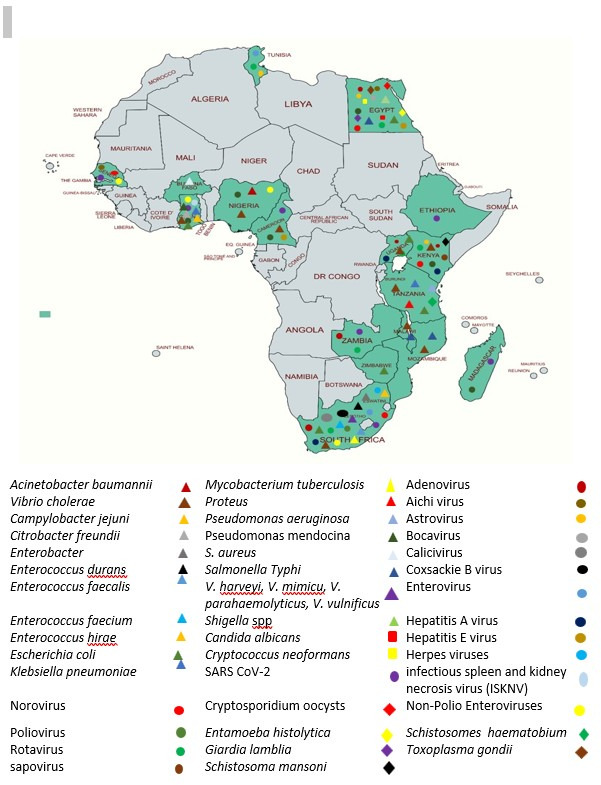
A map of countries in Africa with ES studies showing pathogens identified in each country. The map was retrieved and modified by the authors from https://mapchart.net.

Most ES studies were conducted in urban settings (*n* = 75, 83.3%), while only a few took place in rural areas (*n* = 2, 2.2%). Articles that reported the conduct of ES studies in both rural and urban areas were found to be four (4.4%), including one multi-country study involving Cameroon and Madagascar. However, nine eligible articles did not clearly report the urban or rural nature of their study sites, and some did not specify precise study locations within the country, which resulted in a lower number of studies being categorized in this analysis. Details of the pathogens detected by country in ES studies are presented in Table 2, and Fig. 2 displays a map of Africa, highlighting the countries involved and the corresponding pathogens detected.

### Pathogen detection patterns in environmental surveillance

#### Prevalence of pathogens detected in environmental surveillance studies

Out of the 90 eligible articles reviewed, 14 did not report the sample size used in their analyses. Among the remaining 76 articles, 12 did not clearly state the number of positive detections of pathogens from environmental samples. Therefore, a total of 64 studies were included in the pooled prevalence analysis. These comprised 46 studies on viral pathogens, 154 on bacterial pathogens, 4 on parasitic pathogens, and 1 study on a fungal pathogen. One eligible article (4) reported the detection of bacterial, parasitic, and fungal species and was therefore included in multiple pathogen categories, resulting in a total of 66 entries across these groups. Across the 64 studies, the overall pooled prevalence of pathogen detection in environmental samples was 35.7% (95% CI: 34.9%–36.5%), based on 4,818 positive detections out of 13,502 samples.

#### Prevalence of viruses in environmental samples

Among the 46 studies that focused on viral pathogens, 3,569 viral detections were reported from 7,763 environmental samples, yielding a pooled prevalence of 46.0%. The most commonly investigated viruses were SARS-CoV-2 (10 studies), poliovirus (10 studies), and rotavirus (11 studies), with corresponding pooled prevalence values of 58.4% (1,248/2,137), 38.7% (1,103/2,848), and 58.0% (318/548), respectively. The details of the pooled prevalence of viral pathogens can be found in Table 3.

**TABLE 3:** Prevalence of viruses among eligible ES studies (*n* = 46)

First author	Year	Country	Viral pathogen	No. of detections	Sample size	Prevalence (%) (95% CI)
Grabow et al.	1999	South Africa	Enteroviruses, poliovirus, and coxsackie B virus	174	319	54.5 (49.1–59.9)
Laila et al*.*	2003	Egypt	Poliovirus	74	130	56.9 (48.3–65.1)
Pavlov et al.	2006	South Africa	Poliovirus and NPEV	225	351	64.1 (59.0–68.9)
Kiulia et al.	2010	Kenya	Rotaviruses	22	42	52.4 (37.7–66.6)
Murraye et al.	2013	South Africa	Human calicivirus	42	51	82.4 (69.7–90.4)
Sibanda et al.	2013	South Africa	Hepatitis A virus, rotaviruses, and noroviruses	15	72	20.8 (13.1–31.6)
Abdou et al.	2014	Senegal	Poliovirus and NPEV	209	271	77.1 (71.8–81.7)
Ibrahim et al.	2014	Tunisia	Human enterovirus	52	172	30.2 (23.9–37.5)
Ibrahim et al.	2016	Tunisia	Rotavirus	51	102	50.0 (40.5–59.5)
Ibrahim et al.	2017	Tunisia	Astroviruses	56	102	54.9 (45.2–64.2)
Odoom et al.	2017	Ghana	NPEV	7	36	19.4 (9.8–35.0)
Osuolale et al.	2017	South Africa	Rotavirus	29	70	41.4 (30.6–53.1)
Mabasa et al.	2018	South Africa	Norovirus	78	108	72.2 (63.1–79.8)
Shaheen et al.	2018	Egypt	Astrovirus, norovirus, and rotavirus	6	12	50.0 (25.4–74.6)
Njile et al.	2019	Cameroon	Poliovirus	273	517	52.8 (48.5–57.1)
Van Zyl et al.	2019	Kenya	Pepper mild mottle virus, adenovirus, astrovirus, hepatitis A virus, norovirus, sapovirus, and rotavirus	52	59	88.1 (77.1–94.1)
Rizk et al.	2019	Egypt	Rotavirus	53	72	73.6 (62.3–82.5)
Elmahdy et al.	2019	Egypt	Human adenovirus	68	96	70.8 (61.0–79.1)
El-Senousy et al.	2020	Egypt	Rotavirus	24	24	100 (86.2–100)
Zhou et al.	2020	Kenya	Poliovirus	37	42	88.1 (74.4–96.0)
Kamel et al.	2020	Egypt	Rotavirus	37	42	88.1 (74.4–96.0)
John et al.	2021	Ghana	Poliovirus	3	4	75.0 (30.1–95.4)
Jmii et al.	2021	Tunisia	SARS-CoV-2	19	49	38.8 (26.1–52.9)
Majumdar et al.	2021	Nigeria	NPEV	8	8	100 (67.6–100)
Opere et al.	2021	Kenya	Adenovirus	11	216	5.1 (2.8–8.9)
Kebe et al.	2021	Senegal	Aichi virus	43	66	65.2 (52.5–76.2)
Fagnant-Sperati et al.	2021	Kenya	Poliovirus	73	133	54.9 (46.4–63.1)
Bero et al.	2022	Mozambique	Enteroviruses	25	63	39.7 (28.6–52.0)
Allayeh et al*.*	2022	Egypt	Human adenovirus	64	120	53.3 (44.4–62.0)
Mabasa et al.	2022	South Africa	Human norovirus	162	200	81.0 (74.9–86.0)
Ruhanya et al.	2022	Zimbabwe	Enteroviruses	16	18	88.9 (67.2–96.9)
Johnson et al.	2022	South Africa	SARS-CoV-2	136	272	50.0 (44.1–55.9)
Ernest et al.	2022	Ghana	NPEV and poliovirus type Sabin	35	35	100 (90.1–100)
Ibrahim et al.	2023	Tunisia	SARS-CoV-2 and enterovirus	205	242	84.7 (79.6–88.7)
Fatawou et al.	2023	Cameroon	Hepatitis E virus	3	157	1.9 (0.7–5.5)
Tambe et al.	2023	South Africa	SARS-CoV-2	365	487	74.9 (70.9–78.6)
Saase et al.	2024	Zambia	Human adenovirus and human rotavirus	18	20	90.0 (69.9–97.2)
Abera et al.	2024	Ethiopia	SARS-CoV-2	335	356	94.1 (91.2–96.1)
Shaheen et al.	2024	Egypt	Adenovirus, Aichi virus, bocavirus, and SARS-CoV-2	39	192	20.3 (15.2–26.6)
El-Senousy et al.	2024	Egypt	Human rotavirus	11	33	33.3 (19.8–50.4)
Othman et al.	2024	Tunisia	SARS-CoV-2	27	30	90.0 (74.4–96.5)
Shempela et al.	2024	Zambia	SARS-CoV-2	62	155	40.0 (32.6–47.9)
Salemane et al.	2024	Egypt	Hepatitis E virus	117	536	21.8 (18.5–25.5)
Ewurabena et al.	2024	Ghana	SARS-CoV-2	60	354	16.9 (13.4–21.2)
Ndiaye et al.	2024	Senegal	NPEV	148	281	52.7 (46.8–58.4)
Raharinantoanina et al.	2024	Madagascar	Vaccine-derived poliovirus	0	1,046	0.0 (0.0–0.4)
**Total**				**3,569**	**7,763**	**46.0**

#### Prevalence of bacterial, fungal, and parasitic pathogens

The pooled prevalence of bacterial pathogens was 19.7% (1,074/5,443). The most frequently detected bacterial species were *Escherichia coli* (29.5%, 189/640), *Vibrio cholerae* (22.5%, 581/2,583), and *Salmonella* Typhi (4.1%, 102/2,499). Fungal pathogens had a prevalence of 33.3% (4/12), while parasitic pathogens had a higher pooled prevalence of 60.2% (171/284) (Table 4).

**TABLE 4:** Prevalence of bacterial, parasitic, and fungal pathogens among eligible studies

First author	Year	Country	Pathogen	No. of detections	Sample size	Prevalence (%) (95% Cl)
Madoroba et al.	2010	South Africa	*Vibrio cholerae*	15	594	2.5 (1.5–4.1)
Muchesa et al.	2014	South Africa	Free-living amoeba	150	172	87.2 (81.4–91.4)
Hassan et al.	2024	Egypt	Multi-pathogen (*Campylobacter jejuni, Escherichia coli, Salmonella* Typhi, *Shigella* spp*., Candida albicans, Cryptococcus neoformans, Entamoeba histolytica, Giardia lamblia, Cryptosporidium* oocysts*,* and *Toxoplasma gondii* [sporulated oocysts])	193	245	78.8 (73.2–83.4)
Debes et al.	2016	Cameroon	*Vibrio cholerae*	244	1,011	24.1 (21.6–26.9)
Fri et al.	2017	South Africa	*Vibrio cholerae*	0	80	0.0 (0.0–4.6)
Osuolale et al.	2017	South Africa	*Escherichia coli*	29	70	41.4 (30.6–53.1)
Yirenya-Tawiah et al.	2018	Ghana	*Vibrio cholerae*	267	320	83.4 (79.0–87.1)
Bwire et al.	2018	Uganda	*Vibrio cholerae*	37	322	11.5 (8.5–15.4)
Rizk et al.	2019	Egypt	*Cryptosporidium*	53	72	73.6 (62.4–82.4)
Gildas et al.	2019	Tanzania	*Vibrio cholerae*	15	120	12.5 (7.7–19.6)
Maje et al.	2020	South Africa	*Vibrio cholerae*	3	136	2.2 (0.8–6.3)
Mtetwa et al.	2022	South Africa	*Mycobacterium tuberculosis*	12	12	100 (75.7–100)
Addae-Nuku et al.	2022	Ghana	Multi-bacterial *(Escherichia coli, Klebsiella pneumoniae, Citrobacter freundii, Alcaligenes faecalis,* and *Pseudomonas mendocina)*	123	294	41.8 (36.3–47.5)
Pennance et al.	2022	Tanzania	Schistosomes	21	112	18.8 (12.6–27.0)
Odih et al.	2023	Nigeria	*Acinetobacter baumannii*	77	24	31.2 (21.0–41.5)
Uzzell et al.	2024	Malawi	*Salmonella* Typhi	34	1,127	3.0 (2.2–4.2)
Uzzell et al.	2024	Malawi	*Salmonella* Typhi	34	1,127	3.0 (2.2–4.2)
Gomi et al.	2024	Uganda	*Escherichia coli*	20	31	64.5 (46.9–78.9)

### Methodological approaches in environmental surveillance studies

#### Sampling strategies

Among the 90 eligible articles, two main sampling methods were identified: grab water and Moore swab. The majority of the eligible articles used only the grab water method (*n* = 85, 94.5%), while two used only the Moore swab method. Both methods were used in three (3.3%) studies to collect samples.

#### Concentration techniques

The included articles employed varied laboratory processing and detection methods. Among the laboratory concentration methods, the bag-mediated filtration system, two-phase separation method, skimmed milk flocculation, beef extract solution with aluminum chloride and polyethylene glycol method, as well as filtration and glass wool adsorption-elution methods, were used to concentrate samples in at least five of the eligible articles.

Filtration method (*n* = 30, 33.3%) was used by most of the eligible articles (*n* = 90) to concentrate samples. Among the studies that detected bacteria (*n* = 28), filtration was used to process samples in 11 of the studies (39.3%).

Studies that surveyed for viruses (*n* = 59) were processed by a bag-mediated filtration system in 6 studies (10.2%), two-phase separation method in 22 studies (37.3%), skimmed milk flocculation in 5 studies (8.5%), beef extract solution with AlCl and polyethylene glycol method in 10 studies (16.9%), filtration in 18 studies (30.5%), and glass wool adsorption-elution methods in 7 studies (11.9%). Several included studies employed two or more concentration methods for sample processing. Consequently, individual studies were counted in multiple method categories, which accounts for the cumulative percentages exceeding 100%.

### Detection and analytical methods

Culturing and isolation of pathogens on suitable media or cell lines were conducted in 41 (45.6%) eligible articles for pathogen detection, of which *Vibrio cholerae* was the pathogen with the highest detection (*n* = 11, 26.8%). Different types of PCR techniques such as real-time PCR (RT-PCR), reverse transcriptase-PCR, quantitative-PCR, and others were used in 85 (94.4%) of the eligible articles, and 41 (45.6%) of these eligible articles were sequenced to identify genotype or serotype presence, virulence genes, and AMR genes. AMR genes among pathogens were identified in eight (8.9%) eligible articles. Details of the laboratory processing and detection methods can be found in Table 5.

**TABLE 5:** Sampling, concentration, and detection methods or techniques identified in the eligible articles

Methods or techniques (number of articles [*n,* %])	Type of pathogen (number of articles)	Pathogens of key public health interest that were identified
Sample collection method		
Grab water only, 85 (94.5%)	Viruses (*n* = 58), bacteria (*n* = 24), and parasites (*n* = 3)	Poliovirus (67), SARS-CoV-2 (50), *Vibrio cholerae* (39), and *Schistosoma* (65, 69)
Moore swab only, 2 (2.2%)	Viruses (*n* = 1) and bacteria (*n* = 1)	SARS CoV-2 (63) and *Vibrio cholerae* (84)
Moore swab and grab water, 3 (3.3%)	Bacteria (*n* = 3)	*Salmonella* Typhi (23, 87) and *Vibrio cholerae (39)*
Concentration methods		
Bag-mediated filtration system, 6 (6.7%)	Viruses (*n* = 6)	Poliovirus (67), enterovirus (45), adenovirus (51), SARS-CoV-2 (50), astrovirus (88), hepatitis A virus (88), norovirus (88), sapovirus (88), and rotavirus (88)
Filtration, 30 (33.3%)	Bacteria (*n* = 11)	*Campylobacter jejuni* (56), *Escherichia coli* (56), *Salmonella* Typhi (56), *Shigella* (56), and *Vibrio cholerae* (64)
	Viruses (*n* = 18)	Hepatitis A virus (38), rotaviruses (89), and noroviruses (90)
	Parasites (*n* = 2)	*Entamoeba histolytica* (56), *Giardia lamblia* (56), *Cryptosporidium* oocysts (55), and *Toxoplasma gondii* (56)
	Fungi (*n* = 1)	*Candida albicans* (56) and *Cryptococcus neoformans* (33)
Two-phase separation, 22 (24.4%)	Viruses (*n* = 22)	Adenovirus (91), poliovirus (68), rotavirus (67), and SARS-CoV-2 (21)
Skimmed milk flocculation, 5 (5.6%)	Viruses (*n* = 5)	Norovirus (92), enteroviruses (45), adenovirus (51), rotavirus (51), SARS-CoV-2 (50), and hepatitis E virus (93)
Beef extract solution with AlCl and polyethylene glycol, 10 (11.1%)	Viruses (*n* = 10)	Norovirus (20), enteroviruses (74), and adenovirus (19, 30, 50, 94)
Glass wool adsorption-elution, 7 (7.8%)	Viruses (*n* = 7)	Enteroviruses (37), poliovirus (37), and coxsackie B virus (37),
Pathogen detection method		
Culture and isolation, 41 (45.6%)	Bacteria (*n* = 23)	*Acinetobacter baumannii* (85), *Escherichia coli* (73), *Salmonella* Typhi (56) , *Vibrio cholerae* (64), and *Pseudomonas aeruginosa* (81)
	Viruses (*n* = 18)	Poliovirus (95), coxsackie B virus (37), NPEV (96), and rotavirus (89),
PCR, 85 (94.4%)	Bacteria (*n* = 23), viruses (*n* = 59), and parasites (*n* = 4)	All the pathogens identified except *Candida albicans* (56), *Cryptococcus neoformans* (56)*, Entamoeba histolytica* (56), *Giardia lamblia* (56), *Cryptosporidium* oocysts (56), and *Toxoplasma gondii* (56)
Sequenced, 41 (45.6%)	Bacteria (*n* = 9)	*Escherichia coli* (73), *Vibrio cholerae* (97), and *Pseudomonas aeruginosa* (81)
	Viruses (*n* = 30)	Poliovirus (98), adenovirus (74), and SARS-CoV-2 (21)
	Parasites (*n* = 4)	*Schistosoma haematobium* (65)

#### Pooled prevalence of the frequently detected pathogens with sampling and concentration methods

An analysis focusing on the six most frequently reported pathogens (SARS-CoV-2, poliovirus, rotavirus, *Escherichia coli*, *Vibrio cholerae*, and *Salmonella* Typhi), based on 46 studies, yielded a slightly lower pooled prevalence of 31.5% (95% CI: 30.6%–32.6%) compared to the overall pooled estimate. Sampling and concentration methods for pathogen detection for the six frequently detected pathogens were recorded and can be found in Table 6.

**TABLE 6:** Pooled prevalences of frequently detected pathogens among ES studies with sampling and processing methods used for their detection

First author	Year	Country	Pathogen type	Prevalence of pathogens (%) (no. of positive samples/no. of samples tested)	Pooled prevalence (%) (lower Cl [%]–upper Cl [%])	Sampling method	Concentration method	Detection method
Jimi et al.	2021	Tunisia	SARS-CoV-2	38.8 (19/49)	58.4 (56.3–60.6)	Grab water	Filtration, beef extract, AlCl, polyethylene glycol, and AlOH adsorption and precipitation method-elution	RT-PCR
Johnson et al.	2022	South Africa	SARS-CoV-2	50.0 (136/272)		Grab water	Ultracentrifugation	RT-PCR
Ibrahim et al.	2023	Tunisia	SARS-CoV-2	84.7 (205/242)		Grab water	Filtration, beef extract, AlCl, and polyethylene glycol	RT-PCR
Tambe et al.	2023	South Africa	SARS-CoV-2	74.9 (365/487)		Grab water	Not stated	RT-PCR
Abera et al.	2024	Ethiopia	SARS-CoV-2	941 (335/356)		Moore swab	Not stated	RT-PCR
Shaheen et al.	2024	Egypt	SARS-CoV-2	20.3 (39/192)		Grab water	Two-phase separation, bag-mediated sampling, and skimmed milk flocculation	RT-PCR
Othman et al.	2024	Tunisia	SARS-CoV-2	90.0 (27/30)		Grab water	Filtration	RT-PCR
Shempela et al.	2024	Zambia	SARS-CoV-2	40.0 (62/155)		Grab water	Two-phase separation, bag-mediated sampling, and skimmed milk flocculation	RT-PCR
Ewurabena et al.	2024	Ghana	SARS-CoV-2	16.9 (60/354)		Grab water	Two-phase separation	RT-PCR, pathogen cultured and isolated
Grabow et al.	1999	South Africa	Poliovirus	54.5 (174/319)	38.7 (37.0–40.5)	Grab water	Glass wool adsorption-elution	RT-PCR, pathogen cultured and isolated
Laila et al.	2003	Egypt	Poliovirus	56.9 (74/130)		Grab water	Two-phase separation	RT-PCR, pathogen cultured and isolated
Pavlov et al.	2006	South Africa	Poliovirus	64.1 (225/351)		Grab water	Two-phase separation	RT-PCR, pathogen cultured and isolated
Abdou et al.	2014	Senegal	Poliovirus	77.1 (209/271)		Grab water	Two-phase separation	RT-PCR, pathogen cultured and isolated
Njile et al.	2019	Cameroon	Poliovirus	52.8 (273/517)		Grab water	Two-phase separation	RT-PCR, pathogen cultured and isolated
Zhou et al.	2020	Kenya	Poliovirus	88.1 (37/42)		Grab water	Bag-mediated samples and two-phase separation	RT-PCR, pathogen cultured and isolated
John et al.	2021	Ghana	Poliovirus	75.0 (3/4)		Grab water	Two-phase separation	RT-PCR, pathogen cultured and isolated
Fagnant et al.	2021	Kenya	Poliovirus	54.9 (73/133)		Grab water	Bag-mediated samples and two-phase separation	RT-PCR
Ernest et al.	2022	Ghana	Poliovirus	100 (35/35)		Grab water	Two-phase separation	RT-PCR, pathogen cultured and isolated
Raharinantoanina et al.	2024	Madagascar	Poliovirus	0 (0/1,046)		Grab water	Two-phase separation and ultracentrifugation	RT-PCR, pathogen cultured and isolated
Kiulia et al.	2010	Kenya	Rotavirus	52.4 (22/42)	58.0 (53.9–62.0)	Grab water	Two-phase separation and glass wool adsorption-elution	RT-PCR
Sibanda et al.	2013	South Africa	Rotavirus	20.8 (15/72)		Grab water	Ultrafiltration and glass wool adsorption-elution	RT-PCR
Ibrahim et al.	2016	Tunisia	Rotavirus	50.0 (51/102)		Grab water	Filtration, beef extract, AlCl, and polyethylene glycol	RT-PCR
Osuolale et al.	2017	South Africa	Rotavirus	41.4% (29/70)		Grab water	Filtration and glass wool adsorption-elution	RT-PCR
Shaheen et al.	2018	Egypt	Rotavirus	50.0 (6/12)		Grab water	Ultrafiltration, beef extract, AlCl, polyethylene glycol, and ultracentrifugation	RT-PCR
Van Zl et al.	2019	Kenya	Rotavirus	88.1 (52/59)		Grab water	Bag-mediated samples, filtration, and glass wool adsorption-elution	RT-PCR
Rizk et al.	2019	Egypt	Rotavirus	73.6 (53/72)		Grab water	Filtration and ultracentrifugation	RT-PCR
El-Senousy et al.	2020	Egypt	Rotavirus	100 (24/24)		Grab water	AlOH adsorption and precipitation-elution method and beef extract	RT-PCR
Kamel et al.	2020	Egypt	Rotavirus	88.1 (7/42)		Grab water	AlOH adsorption and precipitation method-elution and beef extract	RT-PCR
Saasa et al.	2024	Zambia	Rotavirus	90.0 (18/20)		Grab water	Bag-mediated samples, two-phase separation, and skimmed milk flocculation	RT-PCR
El-Senousy et al.	2024	Egypt	Rotavirus	33.3 (11/33)		Grab water	Not stated	RT-PCR
Madoroba et al.	2010	South Africa	*Vibrio cholerae*	2.5 (15/594)	22.5 (20–24.1)	Moore swab and grab water	Enriched in alkaline peptone water	RT-PCR, pathogen cultured and isolated
Debes et al.	2016	Cameroon	*Vibrio cholerae*	24.1 (244/1,011)		Grab water	Filtration	RT-PCR, pathogen cultured and isolated
Fri et al.	2017	South Africa	*Vibrio cholerae*	0 (0/80)		Grab water	Filtration	RT-PCR, pathogen cultured and isolated
Yirenya-Tawiah et al.	2018	Ghana	*Vibrio cholerae*	83.4 (267/320)		Grab water	Filtration	pathogen cultured and isolated
Bwire et al.	2018	Uganda	*Vibrio cholerae*	11.5 (37/322)		Grab water	Filtration	RT-PCR, pathogen cultured and isolated
Gildas et al.	2019	Tanzania	*Vibrio cholerae*	12.5 (15/120)		Grab water	Filtration	RT-PCR, pathogen cultured and isolated
Maje et al.	2020	South Africa	*Vibrio cholerae*	2.2 (3/136)		Grab water	Filtration	RT-PCR, pathogen cultured and isolated
Hassan et al.	2017	Egypt	*Escherichia coli*	6.9 (17/245)	29.5 (25.8–33.5)	Grab water	Filtration	pathogen cultured and isolated
Osuolale et al.	2017	South Africa	*Escherichia coli*	41.4 (9/70)		Grab water	Filtration and glass wool adsorption-elution	RT-PCR, pathogen cultured and isolated
Addae-Nuku et al.	2022	Ghana	*Escherichia coli*	41.8 (123/94)		Grab water	Not stated	pathogen cultured and isolated
Gomi et al.	2024	Uganda	*Escherichia coli*	64.5 (20/31)		Grab water	Not stated	RT-PCR, pathogen cultured and isolated
Hassan et al.	2017	Egypt	*Salmonella* Typhi	13.9 (34/245)	4.1 (3.4–5.0)	Grab water	Filtration	pathogen cultured and isolated
Uzzell et al.	2024	Malawi	*Salmonella* Typhi	3.0 (34/1,127)		Moore swab and grab water	Filtration	RT-PCR
Uzzell et al.	2024	Malawi	*Salmonella* Typhi	3.0 (34/1,127)		Moore swab and grab water	Filtration	RT-PCR

**Fig 3:**
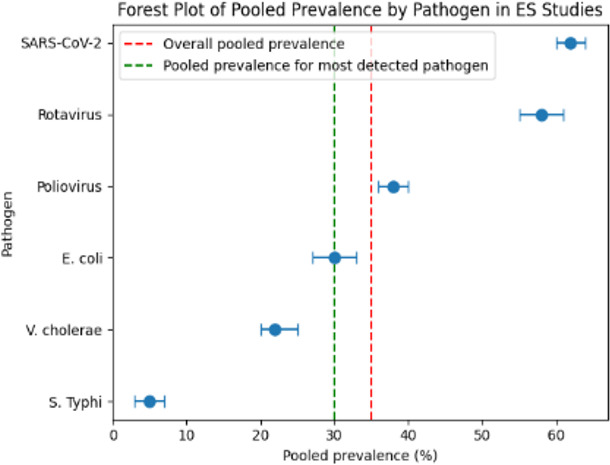
Forest plot of the frequently detected pathogens among the ES studies.

A forest plot was generated to show individual prevalence estimates with corresponding 95% confidence intervals for the six most commonly detected pathogens. A green dashed vertical line representing the pooled prevalence of the six frequently detected pathogens (31.5%) and a red dashed vertical line for the overall pooled prevalence across all studies (35.7%) were included in the plot. The plot illustrated high prevalence values for SARS-CoV-2, poliovirus, and rotavirus, moderate prevalence for *Escherichia coli* and *Vibrio cholerae,* and low prevalence for *Salmonella* Typhi (Fig. 3).

## PUBLIC HEALTH IMPLICATIONS, SURVEILLANCE CAPACITY, AND REGIONAL GAPS IN AFRICA

Environmental surveillance has emerged as a cost-effective, rapid, and reliable approach to detect pathogens and estimate disease burden within communities. The ES approach is particularly valuable at places with limited healthcare access and inadequate diagnostic systems, as it provides early warning signals of possible outbreaks and supports public health interventions (99). Findings from this review indicate that published ES studies have been conducted across most regions in Africa, with most of the papers from the southern and northern areas. South Africa (*n* = 24, 26.7%) and Egypt (*n* = 13, 14.4%) were the African countries with the most recorded number of ES studies on the continent. This aligns with reports that middle-income countries with relatively advanced public health infrastructure, as well as human and research resources, tend to dominate ES studies. These factors were highlighted as the driving force for the numerous ES studies conducted in Brazil (100) and India (101). The robust surveillance network in South Africa, such as the National Institute for Communicable Diseases, has a long history of innovative pathogen monitoring and surveillance studies (102, 103). In addition, the high number of ES studies, especially waterborne disease research studies, conducted in Egypt can partially be driven by the high reliance on the Nile River, which necessitates the continuous water quality monitoring (104), which is comparable to India’s focus on Ganges River water quality surveillance (105). Both South Africa (106) and Egypt (107) have relatively stable economies, enabling substantial investment in research and development. This financial commitment is crucial, as it supports the advanced diagnostic capacity and research infrastructure necessary for effective ES (108, 109).

In contrast, lower-middle-income countries like Ghana (*n* = 8, 8.9%), Nigeria (*n* = 5, 5.6%), and Kenya (*n* = 6, 6.7%) recorded fewer ES studies, potentially reflecting resource constraints, limited technical expertise, challenges in establishing robust ES systems, as well as barriers to publication of results. This pattern is also observed in regions like Southeast Asia, where countries like Bangladesh (110) and Nepal (111) were reported to have few published ES studies despite facing challenges of high infectious disease burden. No ES study was recorded among the eligible papers from the Central African region, and this may reflect infrastructural, financial, and political challenges that hinder surveillance efforts, possibly due to the non-publishing of ES studies from such countries. This regional gap suggests a critical need for targeted investments in capacity building, infrastructure development, and international collaborations to bridge this divide. Furthermore, the absence of ES studies in some regions underscores the importance of enhancing research capacity and funding to strengthen early pathogen detection across the continent (112).

The affordability and relative ease of implementation of ES studies make it a practical tool for public health monitoring in resource-constrained settings (113). Wastewater-based surveillance, an aspect of ES studies, has been highlighted as a cost-effective approach for disease monitoring in low-middle-income countries, offering a viable alternative to traditional clinical surveillance methods (113). The adoption of ES across the African continent remains uneven. This review indicates that fewer than 50% of African countries (*n* = 17, 31.5%) have engaged in and published ES research, limiting the generalizability of findings and highlighting significant gaps in data coverage. Africa is seen to have challenges with surveillance studies, as a systematic review of surveillance systems for antimicrobial resistance in Africa found that only 30 surveillance systems were identified across 47 countries, underscoring the fragmented nature of surveillance efforts (114). Investments in health systems, including surveillance infrastructure, are crucial for improving health outcomes and achieving universal health coverage in Africa (115).

The review provided evidence of the focal viral pathogens that have been studied in many African countries using the ES approach. SARS-CoV-2 (*n* = 14, 23.7%), rotavirus (*n* = 13, 22.0%), and poliovirus (*n* = 12, 20.3%) were reported in most of the eligible articles that detected viral pathogens (*n* = 59). This finding was not surprising, especially for SARS-CoV-2 (116) and poliovirus (117), due to the high public health importance of these two pathogens in Africa and the world as a whole. The establishment of the Global Polio Eradication Initiative (GPEI) by the World Health Assembly in 1988 saw it necessary to encourage countries, especially in Africa, with challenges of acute flaccid paralysis surveillance to complement with the ES approach (15). There is evidence of an ES approach contributing to the successful eradication of poliomyelitis in countries like South Africa (118) and Egypt (119), and the integration of ES into national health policies has been pivotal for continued prevention. There was an expansion of ES programs in South Africa to include 2 sites in each of the 8 metropolitan districts, resulting in a total of 16 ES sites within the country. This expansion aimed to strengthen polio surveillance and maintain the country’s polio-free status (120). Egypt implemented a comprehensive ES system with 46 sewage collection sites across all 27 provinces, integrated into its national polio surveillance framework to ensure early detection and rapid response (121).

During the COVID-19 pandemic, ES gained significant attention as researchers sought solutions to prevent viral transmission and understand the persistence of SARS-CoV-2 in the environment. Wastewater and sewage sampling were widely employed to detect SARS-CoV-2, providing valuable insights into community transmission rates, variant circulation, and viral load dynamics (122). This approach was essential for tracking disease spread and informing public health responses, particularly in the absence of widespread clinical testing. The detection of rotavirus in environmental samples across Africa may be critical, given the severe disease burden it imposes, especially in children. Integrating ES approaches into national surveillance systems can enhance early detection, improve outbreak management, and reduce the overall disease burden in African communities.

*Vibrio cholerae* (*n* = 11, 39.3%) was the most frequently reported bacterial species among the eligible articles (*n* = 28) that reported bacteria, reflecting the ongoing public health burden of cholera in Africa. This is not surprising, as cholera remains a significant public health challenge across the continent, with periodic outbreaks linked to poor sanitation, contaminated water sources, and inadequate waste management (123). Given the high mortality and rapid spread associated with cholera, routine environmental monitoring of *Vibrio cholerae* in water and wastewater is essential for early outbreak detection, risk assessment, and timely public health interventions (123). In contrast, pathogens like *Salmonella* Typhi (*n* = 5, 17.9%)*, Shigella* spp. (*n* = 1, 3.6%), *Mycobacterium tuberculosis* (*n* = 2, 7.1%), and various fungal and parasitic species were less frequently detected in the reviewed studies. This may be due to several factors, including the more stringent resource requirements for detecting these pathogens, subclinical infections that are harder to trace, and the widespread use of antibiotics, which can suppress the detectable levels of these bacteria in environmental samples (124). Additionally, the challenges of detecting these organisms in environmental matrices, coupled with their potentially lower shedding rates compared to cholera, might also contribute to their underrepresentation in ES studies (125).

The two primary sampling methods identified in this review were grab water sampling and the Moore swab method. Grab water sampling emerged as the predominant method in this review, used exclusively in 85 (94.4%) of the eligible studies, compared to 2 (2.2%) studies that relied solely on the Moore swab method. Among the studies focused on specific pathogen groups, grab water sampling was particularly favored, being used in 58 (98.3%) of 59 studies targeting viral pathogens and 24 (85.7%) of 28 studies focused on bacterial pathogens. The widespread use of grab water sampling aligns with the World Health Organization’s (WHO) guidelines, which recommend this approach for the detection of poliovirus in wastewater. Beyond poliovirus, which appeared in 15 (25.9%) of the reviewed studies, this method was also used to detect other significant pathogens like SARS-CoV-2 (*n* = 12, 20.7%) and *Vibrio cholerae* (*n* = 9, 37.5%). The widespread use of grab water sampling can be attributed to its simplicity, cost-effectiveness, and the ease with which it allows for direct quantification of microbial load, given that the volume of collected water is precisely measured. These advantages likely explain its dominance among the studies included in this review.

The Moore swab method, though less commonly used, was applied in this review for the detection of SARS-CoV-2 (63) and *Vibrio cholerae* (84), each in a single eligible study. This method is considered particularly sensitive because it allows for continuous pathogen capture over extended periods, often overnight or longer. This prolonged exposure to flowing wastewater increases the chance of detecting low-abundance or intermittently shed pathogens, making it an effective approach in environments where pathogen concentrations can be highly variable. Previous studies have demonstrated that Moore swabs outperform grab samples in detecting pathogens like SARS-CoV-2, highlighting their suitability for wastewater-based epidemiology, especially in resource-limited settings (126). Studies comparing the effectiveness of the Moore swab and grab water methods for detecting *S.* Typhi (*n* = 2) (23, 87) and *Vibrio cholerae* (*n* = 1) (39) found the Moore swab to be more effective overall (19). Despite its higher sensitivity, the use of the Moore swab in environmental surveillance studies across Africa has been relatively limited. This is partly due to the higher costs associated with its deployment, as it often requires enrichment media to enhance pathogen recovery, and the microbial load estimation can be challenging due to the unknown volume of water absorbed by the gauze (87).

Several methods were employed to process environmental samples across the eligible studies. The most commonly used techniques were filtration (*n* = 30, 33.3%) and two-phase separation (*n* = 22, 24.4%). Among the studies detecting bacterial pathogens (*n* = 28), including *Campylobacter jejuni* (56), *E. coli* (56), *Salmonella* Typhi (56), *Shigella* (56), and *V. cholerae* (64), filtration was used in 11 (36.7%) of these papers. Filtration is particularly effective for bacterial pathogens due to the larger size of these organisms, which enables the use of filter membranes with small pore sizes to effectively capture the bacteria. This method allows for the efficient concentration of pathogens from large water volumes, making it a reliable approach for bacterial detection in environmental samples. Various sample processing techniques were employed across the eligible studies for the detection of viral pathogens (*n* = 59), including poliovirus (67), enterovirus (45), adenovirus (51), SARS-CoV-2 (50), astrovirus (88), hepatitis A virus (88), norovirus (88), sapovirus (88), and rotavirus (88). The most frequently used methods included two-phase separation (*n* = 22, 24.4%), filtration (*n* = 18, 20.0%), and beef extract solution with aluminum chloride and polyethylene glycol (*n* = 10, 11.1%). Other techniques, such as bag-mediated filtration (*n* = 6, 6.7%), skimmed milk flocculation (*n* = 5, 5.6%), and glass wool adsorption-elution (*n* = 7, 7.8%), were also utilized. These processing methods are crucial for concentrating viral particles from large water volumes, thus enhancing the detection sensitivity. The choice of method often depends on factors such as the type of virus, the sample volume, and the specific analytical needs of the study.

The two-phase separation method is a widely recommended sample processing technique by the WHO and has proven effective in ES studies for pathogen detection, particularly for viruses. Among the 59 articles focusing on virus detection, the two-phase separation method was employed in 22 (37.3%) of the studies. This method was used to process samples for various viral pathogens, including adenovirus (91), poliovirus (68), rotavirus (67), and SARS-CoV-2 (21). The two-phase separation involves the use of polyethylene glycol and dextran to create two distinct aqueous phases, facilitating the concentration of viral particles from large water volumes. The method is simple, cost-effective, and uses less hazardous reagents, making it user-friendly for researchers. Additionally, it has been successfully implemented in various regions, including Kenya, in the GPEI Program (127).

Culturing and isolation remain fundamental methods for detecting microbes in environmental samples, particularly for bacteria and certain viruses. Among the eligible studies, 41 (45.6%) used these techniques to identify pathogens such as *Acinetobacter baumannii* (85), *Escherichia coli* (73), *Salmonella* Typhi (56), *Vibrio cholerae* (64), and *Pseudomonas aeruginosa* (81). Despite being recommended by the WHO as the gold standard for the detection of *poliovirus* and *Vibrio cholerae*, these methods are less widely used in many African laboratories (128). This is primarily due to several significant drawbacks, including lower sensitivity compared to molecular techniques, longer processing times, the high cost of specialized culture media or viable cell lines, and the technical expertise required. Additionally, the limited laboratory capacity in many regions further restricts the use of these methods for routine environmental surveillance.

PCR has emerged as a critical tool in water, sewage, and wastewater surveillance, enabling the detection of a broad range of pathogens that are often missed by traditional culturing methods. Its high sensitivity, rapid turnaround time, and straightforward protocol have made it a preferred choice for environmental surveillance, as reflected in its use in the majority of eligible studies (*n* = 85, 94.4%). Pathogens identified using PCR in these studies include *Escherichia coli* (73), *Vibrio cholerae* (97), *Pseudomonas aeruginosa* (81), poliovirus (98), adenovirus (74), SARS-CoV-2 (21), and *Schistosoma haematobium* (65). However, the complex composition of wastewater, coupled with the typically low abundance of target organism DNA and the potential amplification of genes from closely related non-target organisms, can result in both false negatives and false positives in PCR assays, respectively. In some of the studies (*n* = 41, 45.6%), pathogens were further characterized using genomic sequencing to assess circulating genotypes, track transmission pathways, and identify AMR genes. Molecular sequencing has significantly enhanced the ability to detect genetic variations, offering insights into the epidemiology of emerging and re-emerging pathogens. This approach provides critical information for understanding transmission dynamics, identifying potential sources of outbreaks, and monitoring the spread of AMR, ultimately supporting more effective public health responses (129).

A limitation of this review is the potential underestimation of environmental surveillance studies in Africa, as routine wastewater surveillance conducted in some countries may not be published or accessible in the scientific literature search.

## CONCLUSION

Environmental surveillance studies have been conducted and reported in 31.5% (17/54) of African countries from included articles, with the highest number reported in South Africa (*n* = 24, 26.7%), followed by Egypt (*n* = 13, 14.4%). The most commonly targeted pathogens were SARS-CoV-2 (*n* = 14), rotavirus (*n* = 13, 14.4%), and poliovirus (*n* = 12, 13.3%). Grab water sampling was the most frequently used method, featured in 94.4% (*n* = 85) of eligible articles. It was employed in 28.2% (*n* = 24) of studies detecting bacteria and 68.2% (*n* = 58) of those detecting viruses. Two-phase separation (*n* = 22, 37.3%) and filtration (*n* = 11, 39.3%) were the most common sample processing methods for virus (*n* = 59) and bacteria (*n* = 28) detection, respectively. PCR was the dominant pathogen detection method, used in 94.4% (*n* = 85) of the studies.
